# Supplementary material for: Current practice and barriers in the implementation of ultrasound-based assessment of muscle mass in Japan: A nationwide, web-based cross-sectional study
Source: PLoS One. 2022 Nov 3;17(11):e0276855. doi: 10.1371/journal.pone.0276855 (PMC9632777; doi:10.1371/journal.pone.0276855)
Supplement: S1 Fig — Response was obtained across Japan. Response area is colored in this map. This figure was created using R statistical software (version 4.1.0). (DOCX) [file pone.0276855.s001.docx]

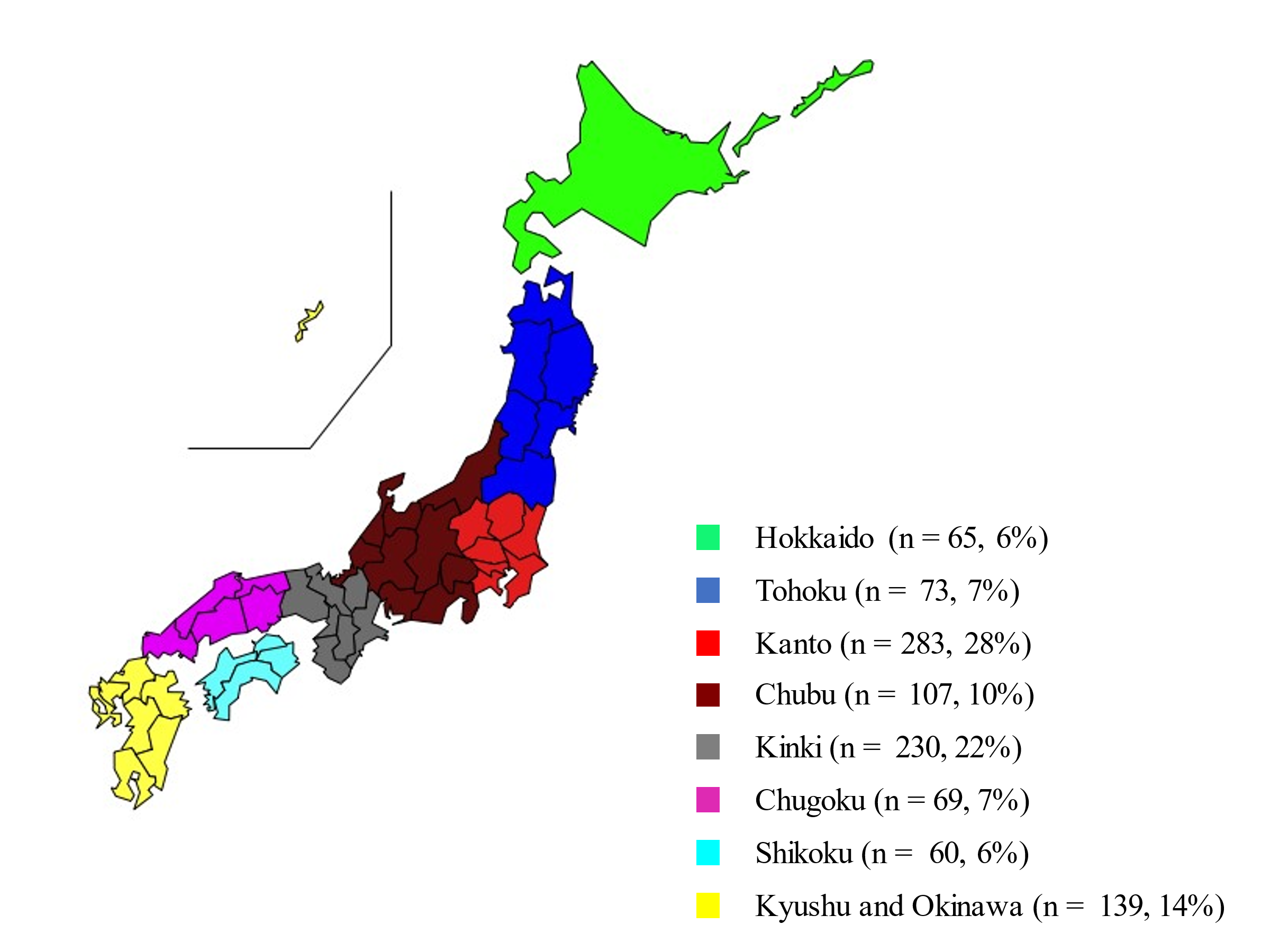


Figure S1. The response distribution in Japan

Response was obtained all across Japan. Response area is colored in this map. This figure was created by using R statistical software (version 4.1.0).
