# Supplementary material for: Current practice and barriers in the implementation of ultrasound-based assessment of muscle mass in Japan: A nationwide, web-based cross-sectional study
Source: PLoS One. 2022 Nov 3;17(11):e0276855. doi: 10.1371/journal.pone.0276855 (PMC9632777; doi:10.1371/journal.pone.0276855)
Supplement: S2 Fig — Response was obtained as shown in the figure. The mailing lists used are shown in different colored arrows. (DOCX) [file pone.0276855.s002.docx]

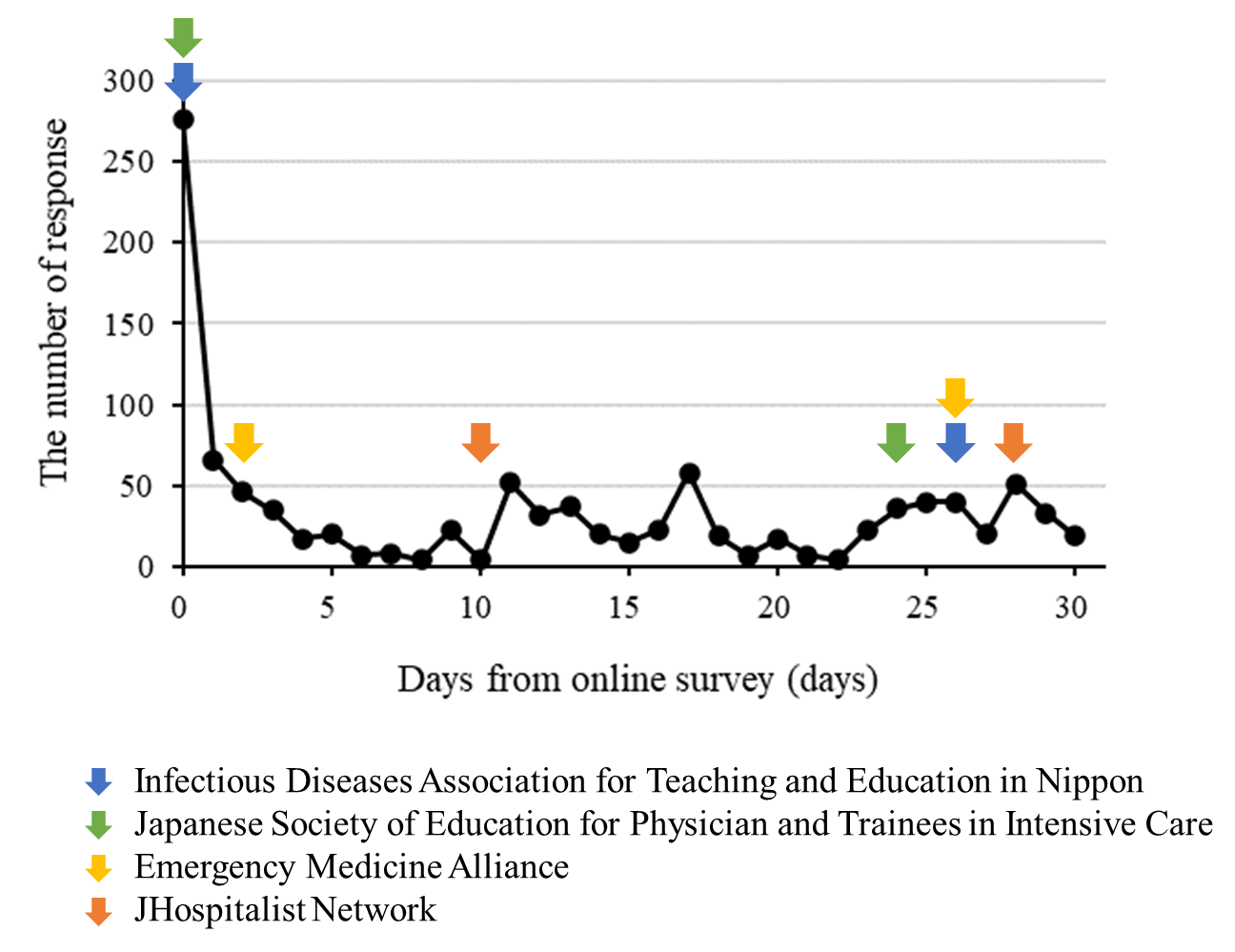


Figure S2. The trend of response in survey period

Response was obtained as shown in this figure. The mailing lists we used were shown in the different colored arrows
