# Supplementary material for: Current practice and barriers in the implementation of ultrasound-based assessment of muscle mass in Japan: A nationwide, web-based cross-sectional study
Source: PLoS One. 2022 Nov 3;17(11):e0276855. doi: 10.1371/journal.pone.0276855 (PMC9632777; doi:10.1371/journal.pone.0276855)
Supplement: S1 Table — (DOCX) [file pone.0276855.s003.docx]

| Table S1. Response distribution among different occupations | | | | | | |
| --- | --- | --- | --- | --- | --- | --- |
| Variables | Overall (n = 1026) | Physician (n = 282) | Physical therapist (n = 489) | Occupational therapist (n = 84) | Nurse (n = 120) | Dietician (n = 51) |
| Hokkaido | 65 (6) | 6 (2) | 29 (6) | 5 (6) | 24 (20) | 1 (2) |
| Tohoku | 73 (7) | 15 (5) | 38 (8) | 11 (13) | 7 (6) | 2 (4) |
| Kanto | 283 (28) | 106 (38) | 114 (23) | 15 (18) | 39 (33) | 9 (18) |
| Chubu | 107 (10) | 36 (13) | 56 (12) | 4 (5) | 8 (7) | 3 (6) |
| Kinki | 230 (22) | 61 (22) | 121 (25) | 17 (20) | 15 (13) | 16 (31) |
| Chugoku | 69 (7) | 18 (6) | 40 (8) | 6 (7) | 1 (1) | 4 (8) |
| Shikoku | 60 (6) | 19 (7) | 13 (3) | 10 (12) | 8 (7) | 10 (20) |
| Kyushu and Okinawa | 139 (14) | 21 (8) | 78 (16) | 16 (19) | 18 (15) | 6 (12) |
| Data were presented in number (percentage). | | | | | | |
