# Supplementary material for: Current practice and barriers in the implementation of ultrasound-based assessment of muscle mass in Japan: A nationwide, web-based cross-sectional study
Source: PLoS One. 2022 Nov 3;17(11):e0276855. doi: 10.1371/journal.pone.0276855 (PMC9632777; doi:10.1371/journal.pone.0276855)
Supplement: S2 Table — (DOCX) [file pone.0276855.s004.docx]

| Table S2. Difference of barriers and interests of ultrasound-based assessment among different occupations | | | | | | |
| --- | --- | --- | --- | --- | --- | --- |
| Variables | Overall (n = 1026) | Physician (n = 282) | Physical therapist (n = 489) | Occupational therapist (n = 84) | Nurse (n = 120) | Dietician (n = 51) |
| Barriers to conduct ultrasound-based assessment, n (%) |  |  |  |  |  |  |
| Education |  |  |  |  |  |  |
| Strongly disagree | 13 (1) | 3 (1) | 5 (1) | 2 (2) | 2 (2) | 1 (2) |
| Disagree | 52 (5) | 14 (5) | 25 (5) | 6 (7) | 5 (4) | 2 (4) |
| Neutral | 95 (9) | 19 (7) | 38 (8) | 11 (13) | 15 (13) | 12 (24) |
| Agree | 558 (54) | 135 (48) | 281 (58) | 43 (51) | 71 (59) | 28 (55) |
| Strongly agre**e** | 308 (30) | 111 (39) | 140 (29) | 22 (26) | 27 (23) | 8 (16) |
| Limited staffing |  |  |  |  |  |  |
| Strongly disagree | 57 (6) | 25 (9) | 20 (4) | 1 (1) | 8 (7) | 3 (6) |
| Disagree | 168 (16) | 43 (15) | 83 (17) | 11 (13) | 27 (23) | 4 (8) |
| Neutral | 175 (17) | 33 (12) | 81 (17) | 23 (27) | 25 (21) | 13 (26) |
| Agree | 486 (47) | 147 (52) | 225 (46) | 39 (46) | 52 (43) | 23 (45) |
| Strongly agre**e** | 140 (14) | 34 (12) | 80 (16) | 10 (12) | 8 (7) | 8 (16) |
| No organized protocol |  |  |  |  |  |  |
| Strongly disagree | 29 (3) | 5 (2) | 16 (3) | 4 (5) | 3 (3) | 1 (2) |
| Disagree | 105 (10) | 20 (7) | 63 (13) | 15 (18) | 3 (3) | 4 (8) |
| Neutral | 264 (26) | 37 (13) | 143 (29) | 32 (38) | 30 (25) | 22 (43) |
| Agree | 463 (45) | 154 (55) | 201 (41) | 24 (29) | 65 (54) | 19 (37) |
| Strongly agre**e** | 165 (16) | 66 (23) | 66 (14) | 9 (11) | 19 (16) | 5 (10) |
| Cost such as introducing the equipment |  |  |  |  |  |  |
| Strongly disagree | 121 (12) | 83 (29) | 17 (4) | 0 (0) | 19 (16) | 2 (4) |
| Disagree | 180 (18) | 96 (34) | 34 (7) | 6 (7) | 38 (32) | 6 (12) |
| Neutral | 156 (15) | 39 (14) | 66 (14) | 13 (16) | 20 (17) | 18 (35) |
| Agree | 397 (39) | 47 (17) | 244 (50) | 48 (57) | 34 (28) | 24 (47) |
| Strongly agre**e** | 172 (17) | 17 (6) | 128 (26) | 17 (20) | 9 (8) | 1 (2) |
| Reliability of the method |  |  |  |  |  |  |
| Strongly disagree | 52 (5) | 6 (2) | 34 (7) | 5 (6) | 6 (5) | 1 (2) |
| Disagree | 237 (23) | 40 (14) | 136 (28) | 27 (32) | 24 (20) | 10 (20) |
| Neutral | 397 (39) | 97 (34) | 177 (36) | 36 (43) | 57 (48) | 30 (59) |
| Agree | 268 (26) | 106 (38) | 113 (23) | 12 (14) | 28 (23) | 9 (18) |
| Strongly agre**e** | 72 (7) | 33 (12) | 29 (6) | 4 (5) | 5 (4) | 1 (2) |
| Necessity and Interests, median (interquartile range) |  |  |  |  |  |  |
| The necessity of ultrasound-based assessment | 7 (6–8) | 7 (5–8) | 8 (6–9) | 6 (5–8) | 7 (5–8) | 7 (5–8) |
| The interest on ultrasound-based assessment | 8 (7–10) | 8 (7–10) | 9 (7–10) | 8 (5–9) | 8 (8–10) | 9 (8–10) |
