# Supplementary material for: Current practice and barriers in the implementation of ultrasound-based assessment of muscle mass in Japan: A nationwide, web-based cross-sectional study
Source: PLoS One. 2022 Nov 3;17(11):e0276855. doi: 10.1371/journal.pone.0276855 (PMC9632777; doi:10.1371/journal.pone.0276855)
Supplement: S3 Table — (DOCX) [file pone.0276855.s005.docx]

| Table S3. Difference between participants with or without ultrasound-based muscle mass assessment | | |  |
| --- | --- | --- | --- |
| Variables | Participants with experience (n = 219) | Participants without experience (n = 807) | p value |
| 1. Participant characteristics |  |  |  |
| Sex (Men), n (%) | 189 (86) | 552 (68) | < 0.01 |
| Clinical experience, years | 10 (7–15) | 11 (6–17) | 0.51 |
| Questionnaire response area , n (%) |  |  |  |
| Hokkaido | 8 (4) | 57 (7) | 0.08 |
| Tohoku | 12 (6) | 61 (8) |  |
| Kanto | 52 (24) | 231 (29) |  |
| Chubu | 25 (11) | 82 (10) |  |
| Kinki | 58 (27) | 172 (21) |  |
| Chugoku | 21 (10) | 48 (6) |  |
| Shikoku | 16 (7) | 44 (6) |  |
| Kyushu and Okinawa | 27 (12) | 112 (14) |  |
| Type of hospital, n (%) |  |  |  |
| University hospital | 78 (36) | 191 (24) | < 0.01 |
| Municipal hospital | 128 (59) | 585 (73) |  |
| Others | 13 (6) | 31 (4) |  |
| Number of hospital beds | 440 (168–690) | 400 (198–650) | 0.45 |
| 4. Barriers and interests to conduct ultrasound-based assessment |  |  |  |
| Education |  |  |  |
| Strongly disagree | 2 (1) | 11 (1) | 0.04 |
| Disagree | 18 (8) | 34 (4) |  |
| Neutral | 25 (11) | 70 (9) |  |
| Agree | 121 (55) | 437 (54) |  |
| Strongly agre**e** | 53 (24) | 255 (32) |  |
| Limited staffing |  |  |  |
| Strongly disagree | 9 (4) | 48 (6) | 0.29 |
| Disagree | 39 (18) | 129 (16) |  |
| Neutral | 29 (13) | 146 (18) |  |
| Agree | 107 (49) | 379 (47) |  |
| Strongly agre**e** | 35 (16) | 105 (13) |  |
| No organized protocol |  |  |  |
| Strongly disagree | 8 (4) | 21 (3) | 0.23 |
| Disagree | 31 (14) | 74 (9) |  |
| Neutral | 53 (24) | 211 (26) |  |
| Agree | 94 (43) | 369 (46) |  |
| Strongly agre**e** | 33 (15) | 132 (16) |  |
| Cost such as purchasing the equipment |  |  |  |
| Strongly disagree | 14 (6) | 107 (13) | < 0.01 |
| Disagree | 25 (11) | 155 (19) |  |
| Neutral | 21 (10) | 135 (17) |  |
| Agree | 98 (45) | 299 (37) |  |
| Strongly agre**e** | 61 (28) | 111 (14) |  |
| Reliability of the assessment |  |  |  |
| Strongly disagree | 12 (6) | 40 (5) | < 0.01 |
| Disagree | 59 (27) | 178 (22) |  |
| Neutral | 60 (27) | 337 (42) |  |
| Agree | 69 (32) | 199 (25) |  |
| Strongly agre**e** | 19 (9) | 53 (7) |  |
| The necessity of ultrasound-based muscle mass assessment | 8 (7–10) | 7 (5–8) | < 0.01 |
| The interest on ultrasound-based muscle mass assessment | 10 (8–10) | 8 (7–10) | < 0.01 |
| Muscle interested to assess in ultrasound-based muscle assessment |  |  |  |
| Upper limb | 64 (29) | 250 (31) | 0.68 |
| Thigh muscle | 163 (74) | 534 (66) | 0.02 |
| Lower leg muscle | 122 (56) | 377 (47) | 0.02 |
| Diaphragm | 127 (58) | 349 (43) | < 0.01 |
| Participants who have joined lecture or hands-on seminar | 95 (43) | 63 (8) | < 0.01 |
| Participants who want to join lecture or hands-on seminar | 192 (88) | 596 (74) | < 0.01 |
| The style to join |  |  |  |
| Lecture | 192 (88) | 596 (74) | < 0.01 |
| Hands-on seminar | 190 (87) | 614 (76) | < 0.01 |
