# Supplementary material for: Current practice and barriers in the implementation of ultrasound-based assessment of muscle mass in Japan: A nationwide, web-based cross-sectional study
Source: PLoS One. 2022 Nov 3;17(11):e0276855. doi: 10.1371/journal.pone.0276855 (PMC9632777; doi:10.1371/journal.pone.0276855)
Supplement: S1 Questionnaire — (DOCX) [file pone.0276855.s006.docx]

**Questionnaire for assessing current status regarding ultrasound-based muscle mass assessment.**

Note: This is a web-based questionnaire survey to reveal the current status and barriers for implementing muscle mass assessment (especially ultrasound assessment) in Japan. This survey is targeting healthcare providers such as physicians, nurses, physical therapists, occupational therapists, dietician, and so on who work in a medical facility. This questionnaire consists of 25 questions, and will take around 5 minutes to complete. This survey is designed not to identify individual or the facilities. The results will be reported at some academic conferences or scientific journals. (*Please do not fill out this questionnaire again if you have already filled it out.)

Section 0. <Consent to participate>

1. **Please place a check if you agree with the purpose and content of this survey**

Section 1. <General information>

1. **Please select your sex.**

- Male
- Female

1. **Please select the area where you live.**

- Hokkaido
- Tohoku
- Kanto
- Chubu
- Kinki
- Chugoku
- Shikoku
- Kyushu and Okinawa

1. **Please select your position as a healthcare provider below.**

- Physician
- Nurse
- Physical therapist
- Occupational therapist
- Dietician
- Other ( )

1. **Please enter the years of clinical experience.**

( )

1. **Please select the type of hospital where you work.**

- University hospital
- Municipal hospital
- Other ( )

1. **Please enter the number of beds at your hospital.**

( )

Section 2. <General muscle mass assessment>

1. **Please select muscle mass assessment methods below you know. (*Multiple responses are possible)**

- Dual Energy X-ray Absorptiometry
- Bioelectrical Impedance Analysis
- Computed Tomography
- Ultrasound
- Mid-Upper Arm Circumference
- Nothing
- Other ( )

1. **Please select muscle mass assessment methods below you conduct. (*Multiple responses are possible)**

- Dual energy X-ray absorptiometry
- Bioelectrical impedance analysis
- Computed tomography
- Ultrasound
- Limb circumference
- Nothing
- Other ( )

1. **Please select the reason to conduct muscle mass assessment.**

- Clinical application
- Research application
- Both of the clinical and research application
- Not conducting

1. **Please select the place where you conduct muscle mass assessment. (*Multiple responses are possible.)**

- Outpatients
- Emergency room
- Intensive care unit
- Acute care unit (such as high care unit, stroke care unit, coronary care unit)
- General ward (such as general bed, recovery phase rehabilitation bed)
- Not conducting
- Other ( )
- Section 3. <Ultrasound-based muscle mass assessment>

1. **Have you ever used ultrasound regardless muscle mass assessment?**

- Yes
- No

1. **Are there any ultrasound devices which you can use in your facility?**

- Yes
- No
- I do not know.

1. **Do you know ultrasound-based muscle mass assessment?**

- Yes
- No

1. **How did you know ultrasound-based muscle mass assessment? (*Multiple responses are possible.)**

- Academic conference
- Lecture or seminar
- Academic paper
- Book
- Social Networking Service
- I do not know ultrasound-based muscle mass assessment.
- Other ( )

1. **Have you ever conducted ultrasound-based muscle mass assessment?**

- Yes
- No

1. **What is the purpose of conducting ultrasound-based muscle mass assessment?**

- Clinical application
- Research application
- Both of the clinical and research application
- Not conducting

1. **How did you study ultrasound-based muscle mass assessment? (*Multiple responses are possible.)**

- Academic conference
- Lecture or seminar
- Academic paper
- Book
- Social Networking Service
- I do not know ultrasound-based muscle mass assessment.
- Other ( )

1. **Please select the muscle where you conduct ultrasound-based muscle mass assessment. (*Multiple responses are possible.)**

- Diaphragm thickness
- Biceps brachii muscle thickness
- Biceps brachii muscle cross-sectional area
- Quadriceps femoris muscle thickness
- Rectus femoris muscle cross-sectional area
- Lower leg muscle thickness
- Lower leg muscle cross-sectional area
- Not performing ultrasound-based muscle mass assessment
- Other ( )

1. **Are there any staff who can conduct ultrasound-based muscle mass assessment? The staff does not include the person answering this questionnaire.**

- Yes
- No
- I do not know.
- Section 4. <Barriers and interests to conduct ultrasound-muscle mass assessment>

1. **Do you agree that the cost such as introducing the equipment is the barrier to conduct ultrasound-based muscle mass assessment?**

- Strongly disagree
- Disagree
- Neutral
- Agree
- Strongly agre**e**

1. **Do you agree that the limited staffing or heavy workload is the barrier to conduct ultrasound-based muscle mass assessment?**

- Strongly disagree
- Disagree
- Neutral
- Agree
- Strongly agre**e**

1. **Do you agree that the insufficient education such as no teaching staff or no lecture is the barrier to conduct ultrasound-based muscle mass assessment?**

- Strongly disagree
- Disagree
- Neutral
- Agree
- Strongly agre**e**

1. **Do you agree that the reliability of the assessment such as insufficient evidence is the barrier to conduct ultrasound-based muscle mass assessment?**

- Strongly disagree
- Disagree
- Neutral
- Agree
- Strongly agree

1. **Do you agree that the** **no organized protocol is the barrier to conduct ultrasound-based muscle mass assessment?**

- Strongly disagree
- Disagree
- Neutral
- Agree
- Strongly agre**e**

1. **Do you think it is necessary to conduct ultrasound-based muscle mass assessment?**

(Strongly unnecessary) 0 1 2 3 4 5 6 7 8 9 10 (Strongly necessary)

1. **Are you interested to conduct ultrasound-based muscle mass assessment?**

(Strongly uninterested) 0 1 2 3 4 5 6 7 8 9 10 (Strongly interested)

1. **Please select the muscle where you want to conduct ultrasound-based muscle mass assessment. (*Multiple responses are possible.)**

- Diaphragm thickness
- Upper limb
- Thigh muscle
- Lower leg muscle
- Not conducting ultrasound-based muscle mass assessment
- Other ( )

1. **Have you ever joined** **the lecture or hands-on seminar about ultrasound-based muscle mass assessment?**

- Yes
- No
- I do not know.

1. **Do you want to join the lecture or hands-on seminar about ultrasound-based muscle mass assessment?**

- Yes
- No
- Neither of that.

1. **What kind of style do you prefer to join to learn ultrasound-based muscle mass assessment?**

- Lecture
- Hands-on seminar
- Both of Lecture and Hands-on seminar
- No preference
- Other ( )
