# Supplementary material for: Current practice and barriers in the implementation of ultrasound-based assessment of muscle mass in Japan: A nationwide, web-based cross-sectional study
Source: PLoS One. 2022 Nov 3;17(11):e0276855. doi: 10.1371/journal.pone.0276855 (PMC9632777; doi:10.1371/journal.pone.0276855)
Supplement: Attachment — Submitted filename: 003_Response_20220621.docx [file pone.0276855.s007.docx]

Dear Emily Chenette,

First of all, thank you for offering us a chance to revise our manuscript for *Plos One*. We appreciate the constructive suggestions by a reviewer. We addressed each comment on point-by-point bases. We believe the revised manuscript is improved, and hope this revise lives up to your expectations.

We look forward to hearing from you regarding our submission.

Sincerely,

Nobuto Nakanishi, MD

(on behalf of all the authors)

**Responses to Reviewer #1:**

1. Sarcopenia is a clinical condition characterized by progressive and generalized loss of muscle mass and muscular force, accompanied by an elevated risk of adverse events. The diagnostic criteria require assessment of muscle mass, muscle force and muscle function impairment, all these exams are time-consuming, with limited use in everyday clinical practice. On the other hand, ultrasound measurement of muscle mass impairment can be used as a quick screening test to assess the presence of sarcopenia in elderly patients. The reviewed paper is interesting, Authors analyzed barriers against wider use of this method in clinical practice, trying to identify motivations that prevent healthcare professional to use it correctly.
   a. We appreciate reviewer’s comments. We have been encouraged by reviewer’s positive comments.
2. Introduction:

Overall quality of the text is good, the language is clear and direct, but I would suggest to consider professional editing to remove some grammar issues. This would improve the quality of the work.

For example: page 2, line 55: “The state of decreased muscle mass in termed…” is not correct.

For example: page 3, line 63: “Muscle mass can be assessed by various measures.” the word “measures” is not the exact term that should be used, I would suggest to replace it with “methods”.?

a. We conducted a professional editing by ENAGO. We attached the proof of editing. We hope our revised manuscript meets your expectations.
3. It would be also useful to describe in more detailed way the diagnostic criteria of sarcopenia, that are actually in use.
a. We added more detailed description about the diagnostic criteria of sarcopenia in the background as following.

According to the guidelines of Asian Working Group for Sarcopenia, the diagnosis of sarcopenia is made based on cutoff values for decreased muscle mass, which differ depending on the method used for assessment (DEXA: cutoff for women and men is 5.4 kg/m^2^ and 7.0 kg/m^2^, respectively; BIA: cutoff for women and men is 5.7 kg/m^2^ and 7.0 kg/m^2^, respectively).[5]

5.	Chen LK, Woo J, Assantachai P, Auyeung TW, Chou MY, Iijima K, et al. Asian Working Group for Sarcopenia: 2019 consensus update on sarcopenia diagnosis and treatment. J Am Med Dir Assoc. 2020;21(3):300-7.e2. doi: 10.1016/j.jamda.2019.12.012. PMID: 32033882

4. Materials and Methods:

The study sample is quite good (1058 responders) but is not homogeneous in relation to their professionality (physicians, nurses, etc..).

Only the descriptive analysis was realized, it would be interesting to examine the association between the groups.

For example, as reported in page 5, line 150: “. For the trend analysis, healthcare providers were included, where the number of participants was ≥50”. According to me the aim of the paper was not to analyze the trend, I would suggest to specify what was the primary aim (from my point of view it was the descriptive analysis, the analysis of differences between groups (experienced vs. non-experienced).

a. Thank you for the opportunity to clarify this point. We added the description to clearly show our primary aim that our paper intended to identify current practices and barriers to implementing ultrasound-based muscle mass assessment. Primary analysis was descriptive analysis. Then, the secondary analyses were to seek the difference between experienced and unexperienced. Furthermore, we added the statistical analysis to examine the difference between the groups.

Primary and secondary analyses

The purpose of this study was to identify current practices and barriers to implementing ultrasound-based muscle mass assessment. The primary analysis was to conduct descriptive analyses among all healthcare workers with sufficient population where the number of participants was ≥50. The secondary analysis was to assess the differences among occupations and between participants with experience of ultrasound-based muscle mass assessment and those without it.

5. Materials and Methods:

According to me the analysis of the difference between healthcare professionals that can use ultrasound in their daily practice (physitian, physiotherapists, ets) and those that cannot use it (pharmacists, engineers, dentists) is not appropriate.

I would suggest excluding of these small sub-groups from the analysis as they are not supposed to use the analyzed method in their practice.?

a. We excluded the small sub-groups (pharmacists, engineers, dentists…) from our analysis. We revised the main Figure 1 (Patient flow) in which pharmacists (n = 12), clinical engineer (n = 7), speech therapist (n = 5), and other occupation (n = 7) were excluded. Furthermore, we reanalyzed secondary analysis about difference between participants with or without experience in the total 1026 participants. We totally revised our manuscript regarding the point throughout our manuscript.

Method

We also excluded those healthcare professions with insufficient (<50) participants.

Results

Overall, 1,058 individuals responded to the survey from all across Japan (S1 Fig. and S1 Table). The trend of the total responses during the survey period is summarized in S2 Figure. Of the 1,058 responders, we excluded 1 nonhealthcare provider and 31 insufficient group populations, including 13 pharmacists, 7 clinical engineers, 5 speech therapists, and 6 other occupations (dentist, medical technologist, biological researcher, practitioner in acupuncture and moxibustion, bonesetter, and certified social worker).

6. The questionnaire should be provided, too.
a. We provided all the questionnaire in the supplemental material from page 2 to 8.
7. Results: The results are expressed as frequencies, the tables should report also the results of Likert scales.
a. We provided the results of Likert scale tables in the supplemental file in Table S2.
8. The paper is interesting, but it reports only descriptive analysis of the healthcare professional’s opinions.
a. As mentioned, this is a descriptive analysis to identify current practices and barriers to implementing ultrasound-based muscle mass assessment. We conducted a web-based questionnaire in the population where sarcopenia evaluation is needed. The practice of muscle mass evaluation varied widely among different occupations. Although the necessity of and interest in ultrasound-based assessment was high among most occupations, only 21% of responders had used the procedure. We found lack of education was the most important barrier that must be overcome to widely implement ultrasound-based assessment. We believe this descriptive analysis contributes to the correct management of sarcopenia.
9. I would suggest to revise the paper and to submit it after again, trying to improve the language and to add more results, if possible.
a. We reanalyzed the data, and revised the manuscript. We added more results in the supplemental file. We conducted English editing to improve the language throughout the manuscript.
