# Supplementary material for: Current practice and barriers in the implementation of ultrasound-based assessment of muscle mass in Japan: A nationwide, web-based cross-sectional study
Source: PLoS One. 2022 Nov 3;17(11):e0276855. doi: 10.1371/journal.pone.0276855 (PMC9632777; doi:10.1371/journal.pone.0276855)
Supplement: Attachment — Submitted filename: 001_Response__20220821.docx [file pone.0276855.s008.docx]

Dear Thomas Phillips,

First of all, thank you for offering us a chance to revise our manuscript for *Plos One*. We appreciate the constructive suggestions by a reviewer. We addressed each comment on point-by-point bases. We believe the revised manuscript is improved, and hope this revise lives up to your expectations.

We look forward to hearing from you regarding our submission.

Sincerely,

Nobuto Nakanishi, MD

(on behalf of all the authors)

**Responses to Reviewer #1:**

1. Thank you for the review, according to me the paper was improved
   a. We appreciate reviewer’s comments. We have been encouraged by the reviewer’s positive comment.
2. In this version it can be considered for publication. I would suggest correcting of some grammar errors, still present in the text.
a. We again conducted a professional editing by ENAGO. All changes were underlined. We hope our revised manuscript meets your expectations.
3. In the discussion You should add confrontation of similar papers analyzing situation in other countries.
a. We added confrontation of similar papers analyzing situation in other countries. We added as following.

Consistent with our findings, other surveys on physiotherapists or rheumatologists have also indicated that one of the most common barriers is the lack of training. [18-20]. According to Ellis et al., 76% of non-ultrasound users answered that lack of training was the barrier to using the method [18]. Potter et al. reported that this barrier accounted for 63% of the participants in their questionnaire [19].

Contrary to expectations, approximately 70% of the participants had access to ultrasound equipment, thus indicating that practitioners in Japan can access ultrasound relatively easily. Although access to the equipment acted as the barrier in 56% of non-ultrasound users in a previous study,[18] it may vary from country to country.

Ultrasound-based muscle mass assessment was most frequently conducted by physical therapists (33%), which is consistent with a previous study reporting that 38% of physiotherapists use ultrasound.[18] Moreover, 15% of physicians performed ultrasound-based muscle mass assessment, which is also consistent with a previous study reporting a finding of 17%.[20]

4. In the background I would evidence all the advantages of the method, why it should be used more widely.
a. We added all the advantages of the ultrasound method to explain why it should be used more widely as following.

There are several advantages in the use of ultrasound for muscle mass assessment. Ultrasound is noninvasive and can be employed by various levels of healthcare providers.[16] It can be used continuously to monitor muscle mass because it is available at the bedside. Furthermore, unlike DEXA and BIA, ultrasound can visually assess skeletal muscle mass without being influenced by fluid balance [10].
