# Supplementary material for: Current practice and barriers in the implementation of ultrasound-based assessment of muscle mass in Japan: A nationwide, web-based cross-sectional study
Source: PLoS One. 2022 Nov 3;17(11):e0276855. doi: 10.1371/journal.pone.0276855 (PMC9632777; doi:10.1371/journal.pone.0276855)
Supplement: Attachment — Submitted filename: 001_Response__20221005.docx [file pone.0276855.s009.docx]

Dear Supat Chupradit,

First of all, thank you for offering us a chance to revise our manuscript for *Plos One*. We appreciate the constructive suggestions by a reviewer. We addressed each comment on point-by-point bases. We believe the revised manuscript is improved, and hope this revise lives up to your expectations.

We look forward to hearing from you regarding our submission.

Sincerely,

Nobuto Nakanishi, MD

(on behalf of all the authors)

**Responses to Reviewer #2:**

1. Overall, this paper is important, interesting, and useful both academically and practically, especially in Japan. It was an extensive and detailed survey that showed some interesting results.
   a. We appreciate reviewer’s comments.
2. However, I must admit that throughout the paper the authors were only interested in Japan. What authors should also be aware of is that the paper will be published in an international journal and it should provide more benefits than just Japan. At the very least, the Conclusion and Discussion section should reduce some mention of Japan and try to link the findings made in Japan with research in other countries.
a. We added some statement to provide more benefits out of Japan. We revised discussion and conclusion section as following.

Discussion

Para. 236

According to Ellis et al., 76% of non-ultrasound users answered that lack of training was the barrier to using the method in the international survey [18]. Potter et al. reported that this barrier accounted for 63% of the participants in the United Kingdom [19].

Para. 250

Although access to the equipment acted as the barrier in 56% of non-ultrasound users in a previous study,[18] it may vary from country to country. Indeed, it is a serious problem to ensure ultrasound equipment and its maintenance in developing countries [23].

Para. 261

Although limb circumference can be measured in any country without any specific equipment [24], we need to know limb circumference measurement does not have a good discriminatory power for identifying low skeletal muscle mass because the indirect muscle mass assessment is affected by numerous factors.[25]

Para. 293

Second, this study was conducted in Japan; However, some questionnaire responses were consistent with previous surveys, possibly presenting important suggestions in other countries.

Conclusion

This questionnaire study examined the current practices in and barriers to implementing ultrasound-based muscle mass assessment.

3. Abstract should be compacted. Many details, including various stats, can be eliminated.
a. We compacted the abstract by eliminating many details including various stats as following. The word counts became 243 words from 284 words.

Muscle mass is an important factor for surviving an illness. Ultrasound has gained increased attention as a muscle mass assessment method because of its noninvasiveness and portability. However, data on the frequency of ultrasound-based muscle mass assessment are limited, and there are some barriers to its implementation. Hence, a web-based cross-sectional survey was conducted on healthcare providers in Japan, which comprised four parts: 1) participant characteristics; 2) general muscle mass assessment; 3) ultrasound-based muscle mass assessment; and 4) the necessity of, interest in, and barriers to its implementation. Necessity and interest were assessed using an 11-point Likert scale, whereas barriers were assessed using a 5-point Likert scale, in which “Strongly agree” and “Agree” were counted for the analysis. Of the 1,058 responders, 1,026 participants, comprising 282 physicians, 489 physical therapists, 84 occupational therapists, 120 nurses, and 51 dieticians, were included in the analysis. In total, 93% of the participants were familiar with general muscle mass assessment, and 64% had conducted it. Ultrasound-based muscle mass assessment was performed by 21% of the participants. Necessity and interest scored 7 (6–8) and 8 (7–10), respectively for ultrasound-based muscle mass assessment. The barriers to its implementation included lack of relevant education (84%), limited staff (61%), and absence of fixed protocol (61%). Regardless of the necessity of and interest in ultrasound-based muscle mass assessment, it was only conducted by one-fifth of the healthcare providers, and the most important barrier to its implementation was lack of education.

4. 3-5 keywords need to be added after the Abstract.
a. We added 5 keywords as following.

Keywords

Muscle mass, Sarcopenia, Ultrasound, Dual-energy X-ray absorptiometry, Bioelectrical impedance analysis
